# Repair of ATAAD with a 3D‐printing assisted pre‐windowed coated stent: A case report

**DOI:** 10.1111/jocs.17109

**Published:** 2022-11-15

**Authors:** Fang Hu, Zi‐qian Zhang, Xin‐hui Zheng, Tao Li, Zhao‐xu Deng

**Affiliations:** ^1^ Key Laboratory of Medical lmaging and Artificial Intelligence of Hunan Province Xiangnan University Chenzhou China; ^2^ Chenhou Cognitive Degeneration Brain Disease Early Warning Technology Research and Development Center Affiliated Hospital of Xiangnan University Chenzhou China; ^3^ Department of Radiology The Third People's Hospital of Chenzhou Chenzhou China

**Keywords:** 3D printing technology, acute type A aortic dissection, case report, interventional therapy

## Abstract

Acute type A aortic dissection (ATAAD) is a life‐threatening vascular disease. We report a case of ATAAD treated with interventional therapy using 3D‐printing assisted pre‐windowing coated stent combined with in situ window‐opening technology. There were few complications and the patient experienced an uneventful recovery.

## 1 INTRODUCTION

Acute type A aortic dissection (ATAAD) is a rare and fatal disease, characterized by an intimal tear in the ascending aorta leading to separation of the layers of the aortic wall and creation of a false cavity (lumen).^1^ Unless prompt surgical repair is performed, patients with an ATAAD usually die from complications.^2^ including rupture of the aorta, pericardial tamponade, aortic regurgitation, end‐organ malperfusion, or acute heart failure.^2^, ^3^ In‐hospital mortality for patients managed medically for ATAAD approaches 57% in the current era.^4^

Herein, we present a case of ATAAD in which three‐dimensional (3D) printing technology was used to create a model of the dissection and pre‐windowed stents. In addition, in situ windowing technology was used to perform successful minimally invasive interventional treatment of the disease.

We present the following case in accordance with the CARE reporting checklist

## 2 CASE PRESENTATION

### 2.1 General information

**Figure 1:**
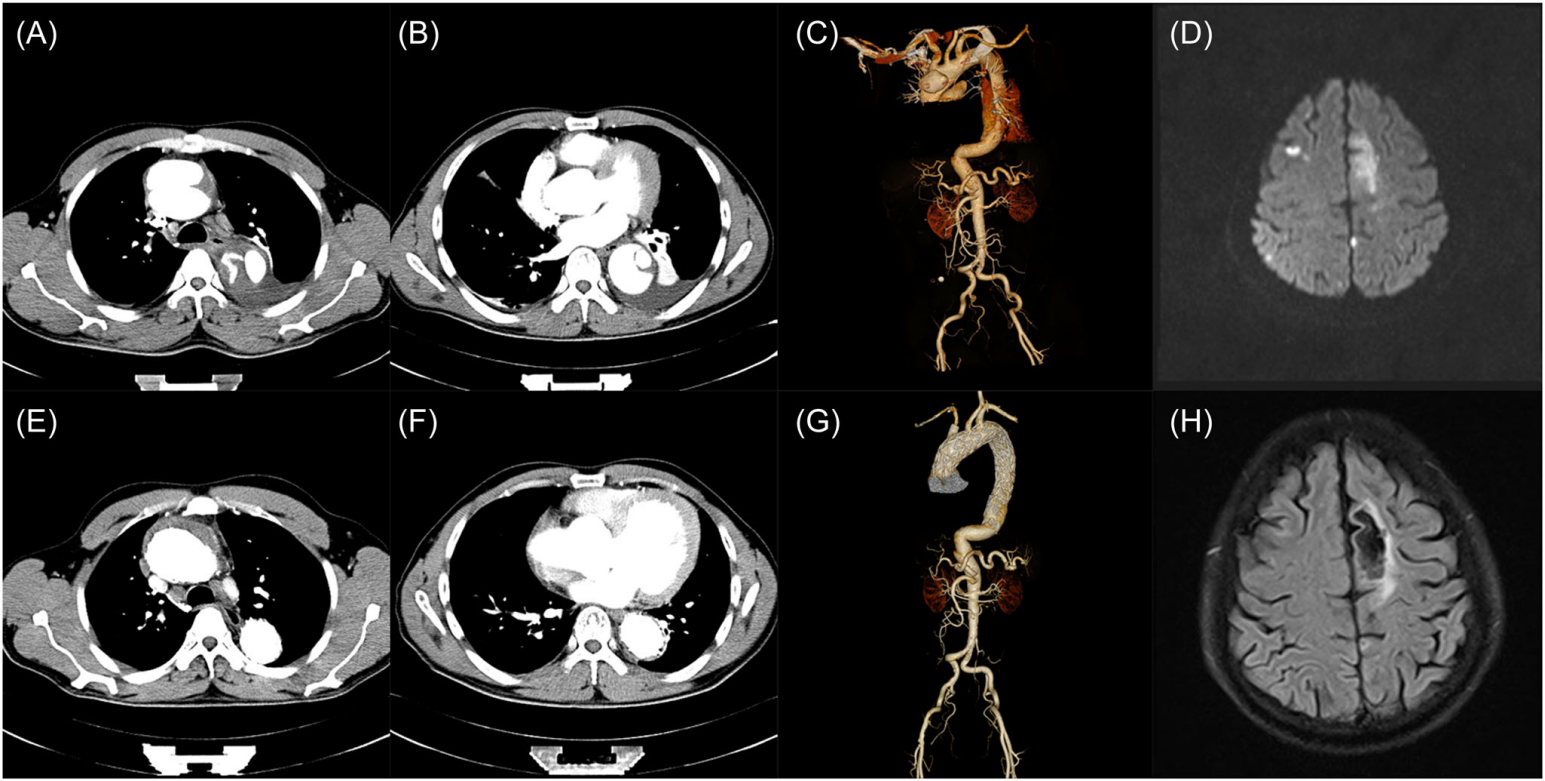
(A) CTA showed a torn diaphragm from the ascending aorta to the left ventricle. (B) Another small laceration at the arch descent, and a false lumen were observed. (C) Three‐dimensional reconstruction CT showed both lesions simultaneously. (d) Postoperative DWI‐MRI of the head suggested multiple acute cerebral infarctions in the brain parenchyma. (E–G) Thoracic aortic CTA 1 year after the operation showed that the dissection flap ended proximally, and the false lumen was obliterated. (H) T_2_‐FLAIR MRI of the head showed that the infarcts had softened. CTA, Computed tomography angiography.

A 43‐year‐old male presented to the emergency department with a 9‐day history of chest and back pain, which was severe and unbearable, accompanied by sweating and unsteady walking, deep breathing can aggravate. Computed tomography angiography (CTA) of the thoracic aorta revealed an ATAAD prompted a torn diaphragm from the ascending aorta to the diaphragm of the left ventricle, and a false lumen was observed, the laceration was located in the ascending aorta, as well as another small laceration at the arch descent, the thoracic aorta was re‐entered, and a slightly high‐density exudation was observed around the aorta (Figure 1A–C). The patient described a history of aortic dissection and hypertension for 5 years.

### 2.2 3D printing process

**Figure 2:**
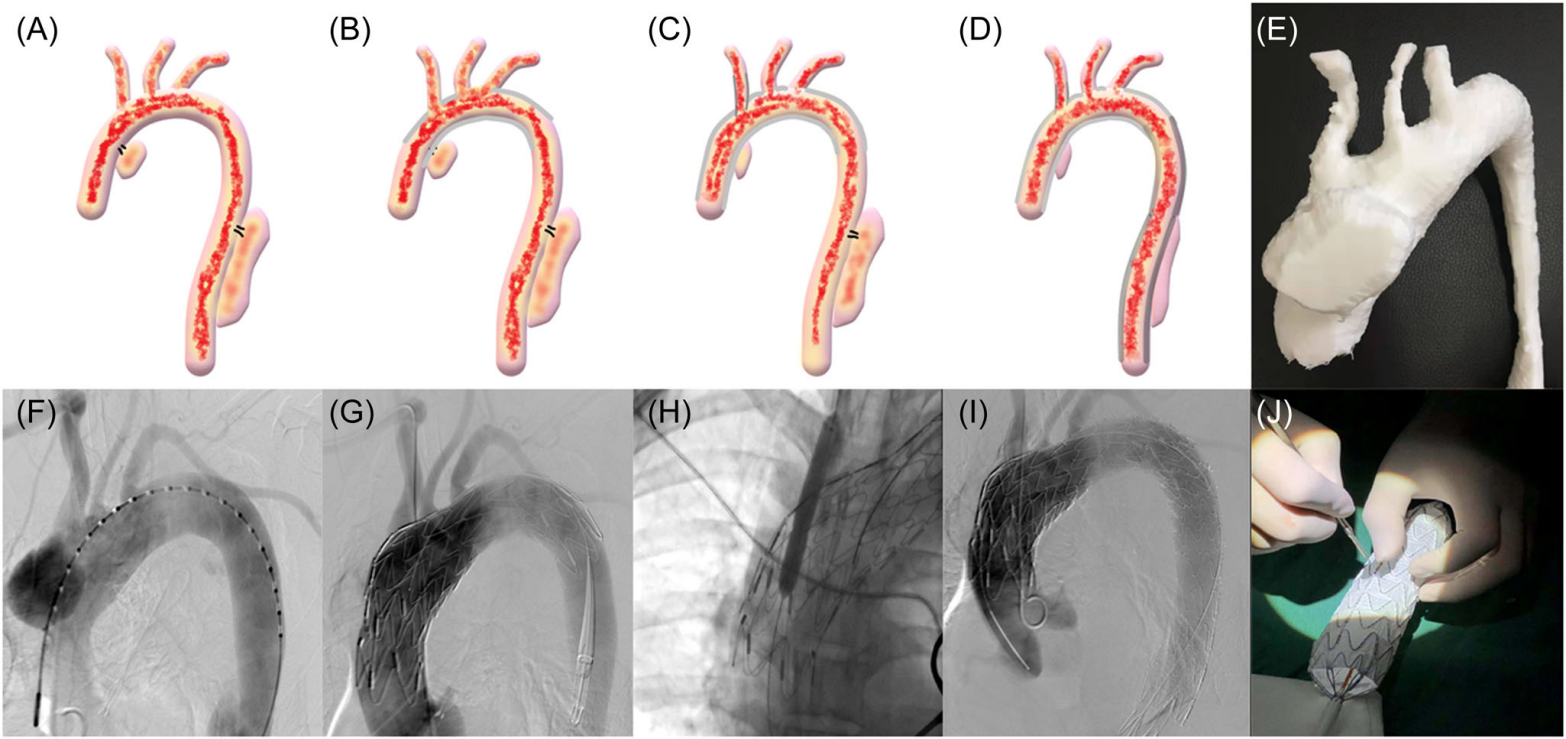
(A, F) Digital subtraction angiography (DSA) revealed a 5.0 × 3.4 cm^2^ dissecting aneurysm in the ascending aorta, as well as a lower thoracic aortic dissection. (B, G) The pre‐windowed coated stent was placed in an appropriate position to occlude the ascending aorta lesion. (C, H) An 8 mm diameter balloon expansion coated stent was used to expand the window opened for the right brachiocephalic artery. (D, I) The last coated stent was placed to close the thoracic aorta rupture. (E) The 3D printed solid 1:1 model of the aortic dissection. (J) A 2 × 4 cm^2^ rectangular window was opened in the stent according to the 3D printed model. DSA of the left common carotid artery and subclavian artery.

MIMICS software was used to process the original CTA data, and then a 1:1 model of the aortic dissection was produced with a prototyping machine (Figure 2F). Preoperative analysis was carried out according to the rapid prototyping model, and accurate classification of aortic dissection was conducted according to the model, so as to fully understand the patient's aortic lesion situation, make the surgical plan, and simulate the operation. According to the measured data, a rectangular window, which was about 2.4 cm*3.5 cm, was opened for the left common carotid artery and the left subclavian artery before the operation (see Figure 2H).

### 2.3 Surgical procedures

Digital subtraction angiography (DSA) displayed the lesions (Figure 2A,F). We put the pre‐windowed coated stent in the appropriate position (Figure 2B,G). The right brachiocephalic artery was not shown on the DSA.

Immediately, in situ windowing for the right brachiocephalic artery was performed; an 8 mm diameter balloon expansion coated stent was used to expand the window. Then, a 14 × 60 mm^2^ film‐coated stent was used to hold the window open (Figure 2C,H). Angiography performed through the humerus artery clearly showed the right brachiocephalic artery.

Finally, the last coated stent was placed to close the thoracic aorta rupture (Figure 2D,I; Supporting Information: Video Legend). The three branches of the aortic arch were visualized by DSA, and there were no signs of stenosis.

Overall, the operation went smoothly without complications. The patient was sent to the intensive care unit (ICU) postoperatively. Care in the ICU consisted of (1) monitoring vital signs, (2) blood pressure reduction, (3) rehydration, and (4) symptomatic supportive treatment.

### 2.4 Postoperative recovery

Postoperative DWI‐MRI of the head suggested multiple acute cerebral infarctions in the brain parenchyma (Figure 1D).

After 1 year, the patient had recovered well from the operation. CTA suggested that the dissection flap ended proximally, and the false lumen was obliterated (Figure 1E–G). MRI of the head showed that the infarcts had softened (Figure 1H).

## 3 DISCUSSION

An ATAAD is a cardiovascular emergency with a high mortality rate, and accounts for 62% of all aortic dissections.^3^ Currently, the primary treatment is thoracotomy and aortic arch replacement. However, the overall early mortality from ATAAD (30‐day or in‐hospital) in patients who receive surgery ranges from 18.9% to 24%.^4^, ^5^

As a result, less invasive endovascular treatment techniques are constantly being explored. However, stent‐graft implantation has been considered a contraindication for type A aortic dissections because of blocking the three branches of the aortic arch. Thus, some researchers have tried using a pre‐windowing coated stent for interventional therapy. The windowing position is usually determined according to the distance measured on two‐dimensional CT images; however, this method is inaccurate and often increases the difficulty and length of the operation due to inaccurate positioning.

In recent years, 3D imaging and printing technology have been widely used in the diagnosis of cardiovascular diseases. The technology allows clinicians to more intuitively understand and determine the 3D relations between aortic dissection and partial branches of the aortic arch. In our patient, a 3D‐printed solid model was used to help determine the specific locations and sizes of the three branches of the aortic arch, and thus to more accurately perform the pre‐windowing of the covered stent. The information from the 3D model helped the operation to be successful. Although the patient developed cerebral infarctions and mild neurological dysfunction, he recovered well.

3D printing technology is useful for the interventional treatment of an ATAAD. The model developed is useful for performing pre‐windowing of the covered stent, and determined the specific locations and sizes of the three branches of the aortic arch and the area of dissection.

## AUTHOR CONTRIBUTIONS

**Zhao‐xu Deng**: Conceptualization; data curation; resources; supervision. **Fang Hu**: Funding acquisition; investigation; project administration; writing – original draft. **Zi‐qian Zhang**: Software; validation; visualization. **Xing‐hui Zheng**: Writing – review & editing; formal analysis. **Tao Li**: Methodology.

## CONFLICT OF INTEREST

The authors declare no conflict of interest.

## ETHICS STATEMENT

This study was approved by the local Research Ethics Review Board of The Third People's Hospital of Chenzhou (China). After the procedures of the operation and study were explained, written informed consent was obtained from the patient.

## Supporting information

The process of implantation of three coated stents in interventional surgery: First, the first pre‐windowed coated stent was implanted to block the dissection lesion of the ascending aorta, then the in‐situ windowing technique was performed, and the second small coated stent was implanted to widen the right brachial artery. Finally, the third coated stent was implanted to block the dissection lesion of the descending aorta.
